# Biological behavior of mesenchymal stem cells on two types of commercial dermal scaffolds: An in vitro study

**DOI:** 10.34172/japid.2024.015

**Published:** 2024-08-11

**Authors:** Omid Moghaddas, Ehsan Seyedjafari, Donya Sadat Mahoutchi

**Affiliations:** ^1^Periodontist, Private Practice, Tehran, Iran; ^2^Department of Biotechnology, College of Science, University of Tehran, Tehran, Iran; ^3^Department of Periodontics, Faculty of Dentistry, Qazvin University of Medical Sciences, Qazvin, Iran

**Keywords:** Acellular dermal matrix, Attachment, Mesenchymal stem cells, Proliferation, Tissue scaffold

## Abstract

**Background.:**

Acellular dermal matrix (ADM) has been introduced as an alternative to autogenous grafts. This study assessed the biological behavior of mesenchymal stem cells (MSCs) on two types of commercial ADM scaffolds.

**Methods.:**

The present in vitro study investigated the behavior of MSCs cultured on scaffold type I CenoDerm® (Tissue Regeneration Corporation) and type II Acellular Dermis (Iranian Tissue Product Co.) as the test groups and an empty well plate as the control group (n=78). Cell attachment was assessed after 12 hours of incubation using 6,4-diamidino-2-phenylindole (DAPI) staining and methyl thiazole tetrazolium (MTT) assay. Cell proliferation was assessed using the MTT assay at 24- and 84-hour and 7-day intervals. Cell morphology was also assessed under a scanning electron microscope (SEM) at 24 hours. MTT assay and DAPI staining were repeated for five samples in all the three groups. Mann-Whitney, ANOVA, and post hoc Tukey tests were used for statistical analysis.

**Results.:**

The DAPI staining and MTT assay showed similar results concerning cell attachment between all the groups at 12 hours (*P*=0.4). At 24 hours, cell proliferation was significantly higher in scaffold groups (*P*<0.001). At seven days, the lowest cell proliferation was noted in the scaffold II group, with a significant difference between the groups (*P*=0.01). At 24 hours, cell expansion was greater in the control group, followed by the scaffold I group.

**Conclusion.:**

Both scaffolds were similar in MSC attachment, but scaffold I appeared superior to scaffold II in terms of MSC proliferation and morphology in vitro.

## Introduction

 Periodontal regenerative treatment aims to provide suitable conditions for periodontal regeneration. Gingival recession can compromise esthetics and lead to root surface caries and tooth hypersensitivity. Several techniques have been suggested for root coverage, including pedicle flap, free gingival graft, guided tissue regeneration, and allografts. Autogenous grafts are procured from the palate or alveolar ridge and have limitations such as donor site morbidity and limited availability.

 Acellular dermal matrix (ADM) is an alternative to autogenous grafts. ADM has applications for root coverage, augmentation of keratinized tissue around teeth and implants, and treatment of gingival recession.^1^ It eliminates the need for autogenous grafts and subsequent pain and discomfort. However, the absence of vasculature and cells in ADM slows down the unity and blending of the graft with the host tissue compared to autogenous grafts. Also, allograft requires cell attachment and anastomosis of the vasculature for maturity and reorganization.^2^

 Tissue engineering enables the fabrication of structures with the desired shape using biomaterials and progenitor cells and also allows cell proliferation and differentiation on suitable scaffolds.^3^ ADM also serves as a temporary matrix for tissue regeneration, enhances the adhesion and proliferation of cells, and plays a key role in the transfer of MSCs to the defect site.^4^

 An ideal scaffold must be biocompatible, easy to use, and easily fixed at the site. Also, it should have interconnected porosities to allow the growth and proliferation of mesenchymal stem cells (MSCs) and angiogenesis. Moreover, it should have osteoconductive and osteoinductive properties.^5^

 The strength and stability of the scaffold also play an important role in the proliferation and differentiation of MSCs.^6^ The size of porosities in the scaffold also affects the attachment, proliferation, and differentiation of MSCs.^7^ Large pores provide less surface for the attachment of cells, and numerous pores increase the number of attachments.^8^

 MSCs are commonly used for cell therapy and tissue engineering due to their self-renewal property and differentiation ability.^9^ These cells can be isolated from different human tissues.^10^

 Many studies are available on MSCs’ attachment, proliferation, and morphology on different commercially available scaffolds; however, studies on the MSCs’ behavior on ADMs produced in Iran are scarce. This study compared the biological behavior of MSCs on two types of commercial ADM scaffolds commonly used for root coverage.

## Methods

 The present in vitro study was conducted on two types of ADM scaffolds, namely scaffold type I (CenoDerm®, Tissue Regeneration Corporation, Tehran, Iran) and scaffold type II (Acellular Dermis, Iranian Tissue Product Co., Tehran, Iran). Of each scaffold, 26 samples were evaluated in this study. Also, 26 empty wells served as controls^11^ (78 samples). One of the samples in each group was used to evaluate the morphologic characteristics of the cells. The scaffolds were coded to blind the operator to the group allocation of scaffolds.

###  Cell isolation and culture 

 MSCs isolated from a sample of the buccal fat pad were seeded and cultured. The tissue specimens were immersed in sterile phosphate-buffered saline (PBS) (Sigma, USA) supplemented with 100-U/mL penicillin (Sigma, USA), 100-μg/mL streptomycin (Sigma, USA), and 2-mg/mL collagenase type IV (Sigma, USA) and incubated at 37°C for 90 minutes. After filtering the cell suspension using a 70-μm filter (SPL, Korea), they were cultured in a 75-cm^2^ cell culture flask (SPL, Korea) containing alpha modification of Eagle’s medium (SPL, Korea) supplemented with 100-μg/mL streptomycin, 15% fetal bovine serum (SPL, Korea), 100-U/mL penicillin, 200-mM L-glutamine and 100-mM ascorbic acid 2-phosphate (Sigma, USA). The cells were incubated with 5% CO_2_ and 95% air at 37 °C for 24 hours. After this period, unattached cells were rinsed off with PBS. The medium was refreshed every three days.

###  Preparation of scaffold and cell seeding 

 Twenty-six rectangular pieces from each scaffold group, measuring 1.5 × 1 cm, with 0.2‒0.6 mm thickness, were rinsed with sterile saline solution (SPL, Korea) in 500-mL flasks for 10 minutes according to the manufacturer’s instructions. The samples were adapted to the bottom of 52 wells in six plates (SPL, Korea). Scaffolds I and II were placed in five wells on each of the five plates. Five empty wells were also considered as the control group in each plate. The sixth plate containing one sample of each scaffold and one empty well as control was used to assess cell morphology. The cell suspension with a density of 16,000 cells/mL was added to the scaffolds and control wells and incubated at 37 °C and 5% CO_2_ for 12, 24, and 84 hours and 7 days. In total, two plates were used for cell attachment assessment using 6,4-diamidino-2-phenylindole (DAPI) staining and methyl thiazole tetrazolium (MTT) assay at 12 hours, and 3 plates were used to assess cell proliferation using the MTT assay at 24 and 84 hours and 7 days.^12^ Five replicates were performed in every assessment at each time interval. One plate was used for cell morphology assessment at 24 hours.

###  Assessment of cell attachment

 DAPI staining: The cell fixation was performed by 12 hours of incubation with 2.5% glutaraldehyde (Sigma, USA) and stained with 50 μg/mL of DAPI stain (Sigma, USA) for 30 minutes. The samples were washed with PBS to eliminate unattached cells. Then, the cells were observed under a fluorescence microscope (TE2000-U; Nikon, Japan) at a 290 nm wavelength and counted in five points (four points at the corners and one at the center).^12^ This was repeated for five samples in all the three groups.

 MTT assay: Optical density (OD) was measured 12 hours after culture to determine the primary attached cells in all the groups with five repetitions.^12^

###  Assessment of cell viability and proliferation 

 The cell viability and proliferation on the scaffolds and the control group were assessed 24 and 84 hours and 7 days after culture using the MTT assay. In this way, 200 μL of RPM1640 and 20 μL of fresh MTT solution (5 mg/mL) (Sigma, USA) were added to the cell culture wells, followed by incubation at 37 °C under 5% CO_2_ for 4 hours.^12^ Tetrazolium salt present in MTT was absorbed by biologically active cells, resulting in formation of purple formazan crystals, which were dissolved by adding isopropanol (Sigma, USA), including 0.1-N HCL (150 mL/well). The OD of the solution was read by a microplate spectrophotometer (SPL, Korea) by decreasing the wavelength from OD690 to OD570.^13^ For assessment of cell proliferation and attachment, cell viabilityat each time interval was performed separately for five samples of each group, and determined based on a linear diagram representing the correlation between OD and cell number.

###  Assessment of cell morphology

 To assess cell morphology, the cells were cultured on scaffolds and a control group and incubated for 24 hours. Then, they were washed twice with PBS, fixed with 2.5% glutaraldehyde for one hour at room temperature, and dehydrated with six graded concentrations of ethanol (from 50% to 100%), and hexamethyldisilazane (Sigma, USA). The samples were then gold-coated and evaluated under a scanning electron microscope (SEM; Nikon, Japan) at × 1000 magnification. One sample of each group was scanned under the SEM. Two parameters were assessed, including scaffold surface area covered with cells (in square micrometers) and roundness of the cells (smaller-to-larger diameter ratio of the cells).^14^ Cell morphology assessment was performed on one sample of each group.

###  Statistical analysis

 Cell proliferation and attachment experiments were performed in five replicates. All the results were statistically analyzed using SPSS 25 (SPSS Inc., USA). Means ± standard deviations were used for adhesion and proliferation data analysis. The Mann-Whitney test was used to statistically analyze cell attachment, while ANOVA was applied to assess the proliferation of cells. Post hoc Tukey tests were applied for pairwise comparisons in cases of significant differences. A *P* value of < 0.05 with a 95% confidence interval was considered statistically significant.

## Results

 Cell attachment was assessed in 30 samples using the MTT assay and DAPI staining (five samples from each group for each test) at 12 hours.

 In the MTT assay, the scaffold II group had the highest cell attachment, followed by the control group. ANOVA showed no significant difference in cell attachment between the three groups (*P* = 0.4).

**Figure 1:**
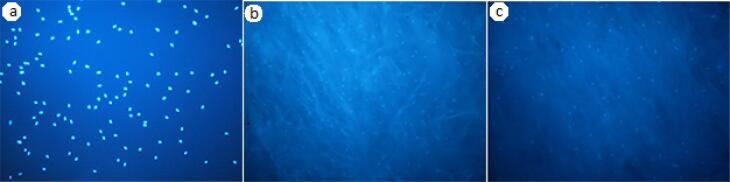
Fluorescence view of DAPI staining at 12 hours for each group. (A) Control group. (B) Scaffold I. (C) Scaffold II

 In DAPI staining (Figure 1), the highest attachment was noted in scaffold I, followed by the control group. ANOVA showed that the difference between the three groups was not significant (*P* = 0.4) (Table 1).

**Table 1:** Cell attachment determined by the MTT assay and DAPI staining in the three groups at 12 hours

**Group/Technique **	**MTT (OD)**	**DAPI (number)**
Control group	0.12 ± 0.011	94.8 ± 13.003
Scaffold I	0.11 ± 0.01	99.9 ± 13.4
Scaffold II	0.13 ± 0.017	90.6 ± 8.4
ANOVA test result	*P* < 0.4	*P* < 0.4

DAPI: 6,4-diamidino-2-phenylindole stain, MTT: methyl thiazole tetrazolium assay, OD: optical density.

 In the assessment of cell proliferation, 45 samples were evaluated at 24 and 84 hours and 7 days in all the groups (5 samples in each group) with MTT assay (Table 2).

**Table 2:** Proliferation rate in the three groups at different time points (MTT assay)

**Group**	**Time**	**Mean and standard deviation (OD)**
Control	24 hours	0.11 ± 0.01
	84 hours	0.3 ± 0.008
	7 days	1.11 ± 0.13
Scaffold I	24 hours	0.14 ± 0.01
	84 hours	0.2 ± 0.0
	7 days	0.58 ± 0.08
Scaffold II	24 hours	0.15 ± 0.01
	84 hours	0.2 ± 0.06
	7 days	0.27 ± 0.06

MTT: methyl thiazole tetrazolium assay, OD: optical density.

 Over time, cell proliferation increased in all the three groups. At 24 hours, the highest proliferation rate was noted in the scaffold II group, followed by scaffold I. ANOVA showed that the proliferation rate was significantly higher in scaffolds I and II groups compared to the control group (*P* < 0.001). Pairwise comparisons by Tukey’s test showed that the difference between the two scaffolds was not significant (*P* = 0.8).

 At 84 hours, the highest proliferation rate was noted in the control group, followed by the scaffold I group. ANOVA showed no significant difference in this regard between the control and scaffold groups (*P* = 0.2) or between the two scaffold groups (*P* = 0.9).

 At seven days, the highest proliferation rate was noted in the control group, followed by the scaffold I group. The difference in this regard between the three groups was statistically significant (*P* = 0.01).

 In all the three groups, the proliferation rate increased over time (shown by the MTT assay) such that in the control group, multiple comparisons revealed significant differences in the proliferation rate over time (*P* < 0.001).

 In scaffold I, ANOVA showed that the difference in the proliferation rate was statistically significant over time (*P* = 0.01). In scaffold II, ANOVA showed that the proliferation rate difference was not significant over time (*P* = 0.2).

 In the three groups, the highest proliferation rate was noted at 84 hours and 7 days (the highest cell count was noted in the control group, with the lowest in the scaffold II group).

**Figure 2:**
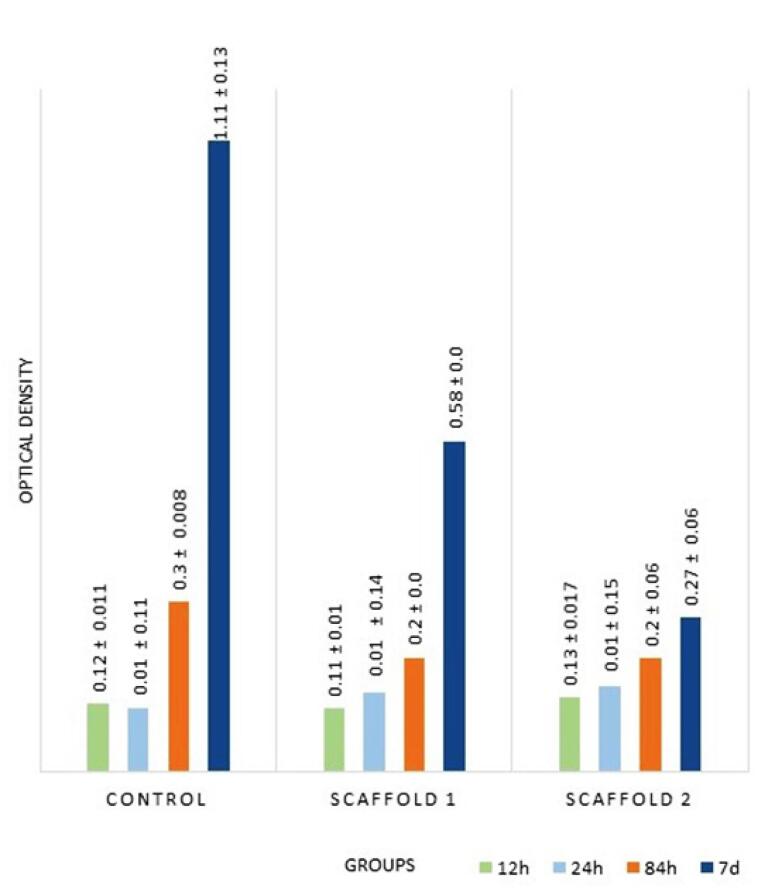
Results of MTT assay (OD) at all time intervals in all groups

**Figure 3:**
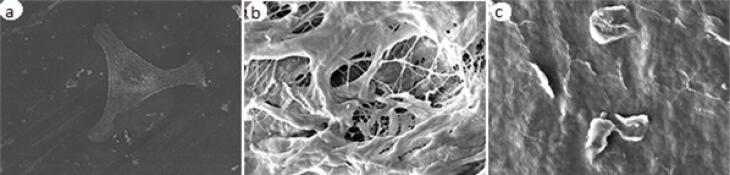
SEM micrograph of MSCs on the scaffold surface at × 1000 magnification 24 hours after culture. (A) Control group. (B) Scaffold I. (C) Scaffold II

Figure 2 shows the OD of MSCs of all the groups at all time intervals. Assessment of cell morphology under SEM at 24 hours revealed greater cell expansion with more appendages in the control group, followed by the scaffold I group compared to the scaffold II group (Figure 3).

## Discussion

 In this study, two commonly used scaffolds, CenoDerm and Acellular Dermis, were used. Attachment (at 12 hours) and proliferation (at 24 and 84 hours and 7 days) of MSCs cultured on these scaffolds were assessed using the MTT assay plus DAPI staining and MTT assay, respectively. Morphological properties of cells were also evaluated under SEM at 24 hours. The results showed no significant difference between these scaffolds concerning cell attachment at 12 hours. However, better results were achieved with CenoDerm at 24 hours and 7 days concerning cell morphological properties and cell proliferation, respectively.

 Cell attachment is the first response of the cell to scaffold.^15^ Primary cell attachment to scaffold depends on the size and amount of porosities, water, and protein absorption^16^ and plays an essential role in the proliferation of cells.

 According to Pabst et al,^17^ an autologous scaffold enhancing proliferation of human gingival fibroblasts, endothelial cells, osteoblasts, and oral keratinocytes in vitro can also show higher angiogenic properties in vivo. Thus, scaffolds might show more favorable behaviors in vitro and have higher applicability in vivo.

 In the present study, the MTT assay showed no significant difference at 12 hours in cell attachment between the three groups. Similarly, Ma et al^18^ used an MTT assay and showed proper attachment of fibroblasts to bilayer dermal equivalent. They indicated that using bilayer dermal equivalent also resulted in optimal regeneration in vivo. Thus, the results of MTT can be generalized to the clinical setting.

 Hussein et al^19^ also assessed the attachment of fibroblasts to scaffolds with different sterilization methods using the MTT assay and DAPI staining, with both tests showing similar results. Thus, DAPI staining can confirm the results of the MTT assay in vitro. Also, the results of DAPI staining in vitro can be generalized to the clinical setting. In the present study, to confirm the results of the MTT assay, DAPI staining was also performed after 12 hours, which showed the same results, and both showed no significant difference between the three groups in cell attachment. Regarding the current study analysis, it might be concluded that the attachment of cells was the same in two types of scaffolds. Thus, they probably have the same efficacy for use in the clinical setting regarding attachment of MSCs.

 SEM showed morphological differences, demonstrating the superiority of scaffold I to scaffold II, which indicates the more biologically active cells.^20^ Greater expansion of cells in the control group might be due to the smoother surface of wells in the control group compared to the porous surface of scaffolds.^21^

 The current study assessed the proliferation of MSCs in the three groups after 24 and 84 hours and 7 days using the MTT assay. It showed that the proliferation rate significantly increased over time, which was not significant in scaffold II between time intervals. At 24 or 84 hours, there were no significant differences between the scaffold groups in this respect. At 7 days, the significantly lowest cell population rate was noted in the scaffold II group. Overall, cell proliferation in the scaffold I group was higher than in the scaffold II group. The higher proliferation rate in the control group at each time interval might be attributed to the smooth surface of the plate compared to the porous surface of scaffolds. As previously confirmed, surface topography affects the attachment and differentiation of cells.^22^ Osteoblasts have a greater attachment to rougher surfaces, while fibroblasts and MSCs better adhere to smoother surfaces.^23-25^

 In assessing the proliferation rate and morphological properties, scaffold I showed better results than scaffold II. Thus, it might also be superior for clinical use because evidence shows that the results of the MTT assay can be generalized to the clinical setting.

 We assessed MSC morphology, attachment, and proliferation, which are essential parameters in wound healing and repair. Using both DAPI and MTT assays simultaneously was a strength of our study. Also, no previous study has compared these two scaffolds concerning MSC behavior. The study was performed blindly, and each measurement was repeated five times.

 Hydrophilicity, pore size, biocompatibility, mechanical properties, composition, and solvent or toxic compounds in the scaffold all affect cell seeding.^13^ The structure of biomaterials in the cellular matrix is also important and affects cell behaviors such as attachment, proliferation, and differentiation.^26^ Attachment and proliferation of cells on scaffolds depend on the availability of nutrients, porosity, and interconnection between pores.^27^ The Strength and density of the scaffold also affect cell morphology.^28^ According to the above, further studies are recommended to compare these scaffold structural properties and the efficacy of these scaffolds in MSCs’ behavior in clinical situations.

## Conclusion

 Both scaffolds showed similar efficacy in attachment of MSCs in vitro, but the proliferation of MSCs after 7 days was higher on scaffold I compared to scaffold II. Also, MSCs on scaffold I were more active, expanded more, and had more cellular appendages. Scaffold I was superior to scaffold II in terms of proliferation and morphology of MSCs in vitro.

## Acknowledgments

 As the authors of this paper, we thank the Nanotechnology Department of Tehran University of Medical Sciences for allowing us to conduct our experiments in their laboratory. We also thank Amirkabir University for SEM analyses.

## Competing Interests

 The authors declare no competing interests.

## Consent for Publication

 Not applicable.

## Data Availability Statement

 All data from the current study are available upon request from the corresponding author.

## Ethical Approval

 Not applicable. The study was conducted in vitro without human participation. Therefore, the study did not have any ethical registrations.
